# Establishment of a resource recycling strategy by optimizing isobutanol production in engineered cyanobacteria using high salinity stress

**DOI:** 10.1186/s13068-021-02023-8

**Published:** 2021-08-30

**Authors:** Xiao-Xi Wu, Jian-Wei Li, Su-Fang Xing, Hui-Ting Chen, Chao Song, Shu-Guang Wang, Zhen Yan

**Affiliations:** 1 Shandong Key Laboratory of Water Pollution Control and Resource Reuse, School of Environmental Science and Engineering, Shandong University, Qingdao, 266237 Shandong China; 2 Suzhou Research Institute, Shandong University, Suzhou, 215123 Jiangsu China

**Keywords:** Isobutanol, High salinity, Cyanobacteria, Recycling

## Abstract

**Background:**

Isobutanol is an attractive biofuel with many advantages. Third-generation biorefineries that convert CO_2_ into bio-based fuels have drawn considerable attention due to their lower feedstock cost and more ecofriendly refining process. Although autotrophic cyanobacteria have been genetically modified for isobutanol biosynthesis, there is a lack of stable and convenient strategies to improve their production.

**Results:**

In this study, we first engineered *Synechococcus elongatus* for isobutanol biosynthesis by introducing five exogenous enzymes, reaching a production titer of 0.126 g/L at day 20. It was then discovered that high salinity stress could result in a whopping fivefold increase in isobutanol production, with a maximal in-flask titer of 0.637 g/L at day 20. Metabolomics analysis revealed that high salinity stress substantially altered the metabolic profiles of the engineered *S. elongatus*. A major reason for the enhanced isobutanol production is the acceleration of lipid degradation under high salinity stress, which increases NADH. The NADH then participates in the engineered isobutanol-producing pathway. In addition, increased membrane permeability also contributed to the isobutanol production titer. A cultivation system was subsequently developed by mixing synthetic wastewater with seawater to grow the engineered cyanobacteria, reaching a similar isobutanol production titer as cultivation in the medium.

**Conclusions:**

High salinity stress on engineered cyanobacteria is a practical and feasible biotechnology to optimize isobutanol production. This biotechnology provides a cost-effective approach to biofuel production, and simultaneously recycles chemical nutrients from wastewater and seawater.

**Supplementary Information:**

The online version contains supplementary material available at 10.1186/s13068-021-02023-8.

## Background

Escalating concerns regarding global warming, air pollution, and fossil fuel scarcity have spurred great interest in biofuel production [1]. Traditional methods of producing biofuels based on microbial cell factories utilizing sugar-based crops and lignocellulosic biomass as feedstocks threaten food security and pose problems for waste disposal [2, 3]. Third-generation biorefineries that can convert atmospheric CO_2_ into bio-based fuels and chemicals have emerged as a promising and effective technology [4, 5]. Commercialized products of third-generation biorefineries include bioethanol, biodiesel, and biobutanol, which are applicable to renewable energy. Among these biofuels, biobutanol is proposed as an attractive substitute for gasoline, given its lower hygroscopicity and vapor pressure, as well as higher octane numbers compared with bioethanol and biodiesel [6].

Studies on biobutanol production originated from a fermentative acetone–butanol–ethanol pathway endogenous to *Clostridium* strains. This pathway has been well-explored and implemented not only in its native host, but also in many other model species for *n*-butanol production [7–10]. Non-fermentative pathways based on amino acid biosynthesis can be genetically modified for various biobutanol productions, such as *n*-butanol, isobutanol and other branched-chain biobutanol [6]. Biosynthesis of isobutanol is achieved by metabolic engineering of valine synthesis in microbes starting in the pyruvate pool. Accordingly, 2-ketoisovalerate, a key intermediate in the valine biosynthesis pathway, is redirected to isobutyraldehyde, followed by isobutanol by introducing a 2-ketoacid decarboxylase and an alcohol dehydrogenase [6, 11]. KivD from *Lactococcus lactis* is a well-characterized enzyme responsible for the decarboxylation of 2-ketoacid into aldehydes, and has the highest specific activity towards 2-ketoisovalerate [12]. Several alcohol dehydrogenases, such as AdhA from *L. lactis*, YqhD from *Escherichia coli* and Adh2 from *Saccaromyces cerevisiae*, were engineered successfully for isobutanol production [13]. To increase the 2-ketoisovalerate flux, acetolactate synthase (AlsS) from *Bacillus subtilis*, ketol-acid reductoisomerase (IlvC) and dihydroxy-acid dehydratase (IlvD) from *E. coli* involved in the valine synthesis pathway were typically introduced into the host [5].

The heterotrophic biosynthesis of isobutanol using an organic carbon source has been implemented into various model microbial cell factories. Versatile metabolic engineering strategies and batch culture technologies have been explored to improve the its titer [6, 14–16]. For example, engineered *E. coli* can produce 50 g/L of isobutanol in a bioreactor with in situ product removal using gas stripping [17]. By overexpressing IlvD and deleting a pyruvate decarboxylase, the metabolic flux was strengthened to 2-ketoisovalerate in an engineered *S. cerevisiae*, producing 143 mg/L of isobutanol [18]. A total of 2.2 g/L of isobutanol was achieved in the engineered *Pichia pastoris* by employing an episomal-plasmid-based expression system to fine-tune the expression of all pathway enzymes [19]. Engineered *B. subtilis* produced up to 2.6 g/L of isobutanol in a flask fed-batch fermentation [20], while engineered *Geobacillus thermoglucosidasius* produced 3.3 g/L of isobutanol at a relatively high temperature 50 °C [21]. However, the autotrophic biosynthesis of isobutanol using CO_2_ as the carbon source has only been engineered into two model cyanobacteria: *Synechococcus elongatus* PCC7942 (hereafter *S. elongatus*) and *Synechocystis* sp. PCC 6803 (hereafter *Synechocystis*). Strategies for improving the production have not been well developed for heterotrophic biosynthesis. Atsumi et al. in 2009 engineered *S. elongatus* to produce up to 450 mg/L of isobutanol [11], while the product titer reach up to 550 mg/L upon diverting a portion of the carbon from glycogen synthesis to isobutanol by deleting a *glgC* gene [22]. In two other studies, *Synechocystis* was engineered to produce 298 mg/L of isobutanol using a mixtrophic culture [23], while Miao et al. in 2018 achieved a cumulative titer of 911 mg/L at day 40 via engineered *Synechocystis* in an HCl-titrated medium [24].

Environmental stresses on microbes are external conditions that impact a variety of physiological functions, in which some are inhibited or lost, while others are enhanced or induced [25, 26]. Environmental stresses from physical and chemical stimulations can enhance the accumulation of microalgal lipids [27, 28]. For example, variations in light conditions can alter the growth and metabolism of marine microalgae, resulting in higher lipid productivity and contents [29]. Nitrogen depletion and high salinity have been shown to trigger lipid accumulation for both marine and freshwater microalgae [30]. The intrinsic mechanism for lipid accumulation under salt stress has been studied extensively. For the marine *Chlamydomonas* sp. JSC4, a precursor for lipid biosynthesis, glycerol-3-phosphate (G3P), was found to be higher under salt stress [31]. For the freshwater *Chlorella sorokiniana* sp. HS1, redirecting carbon flow toward energy-storage is a pivotal reason for lipid accumulation [32]. All of these studies offer attractive strategies and technologies for enhancing biodiesel production via third-generation biorefineries. Nevertheless, not much is known about how environmental stresses impact the biofuel production of engineered cyanobacteria introduced in an external metabolic pathway.

In this study, the cyanobacteria *S. elongatus* was engineered to produce isobutanol by introducing exogenous AlsS, IlvC, IlvD, KivD, and AdhA. It was found that high salinity stress significantly enhanced isobutanol production, while nitrogen starvation did not. Comprehensive analysis of the metabolic profiles of engineered *S. elongatus* under high salinity condition suggest that increased abundance of NADH due to accelerated lipid degradation was the major reason for enhanced isobutanol production. In addition, increased membrane permeability also contributed to the isobutanol production titer. A cultivation system was then developed by mixing synthetic wastewater with seawater to produce isobutanol, and its feasibility was demonstrated. This study thus proposes a novel biotechnology approach to producing isobutanol in a cost-effective and ecofriendly manner.

## Results and discussion

### Exposure to high salinity stimulates engineered *S. elongatus* to produce isobutanol

**Fig. 1:**
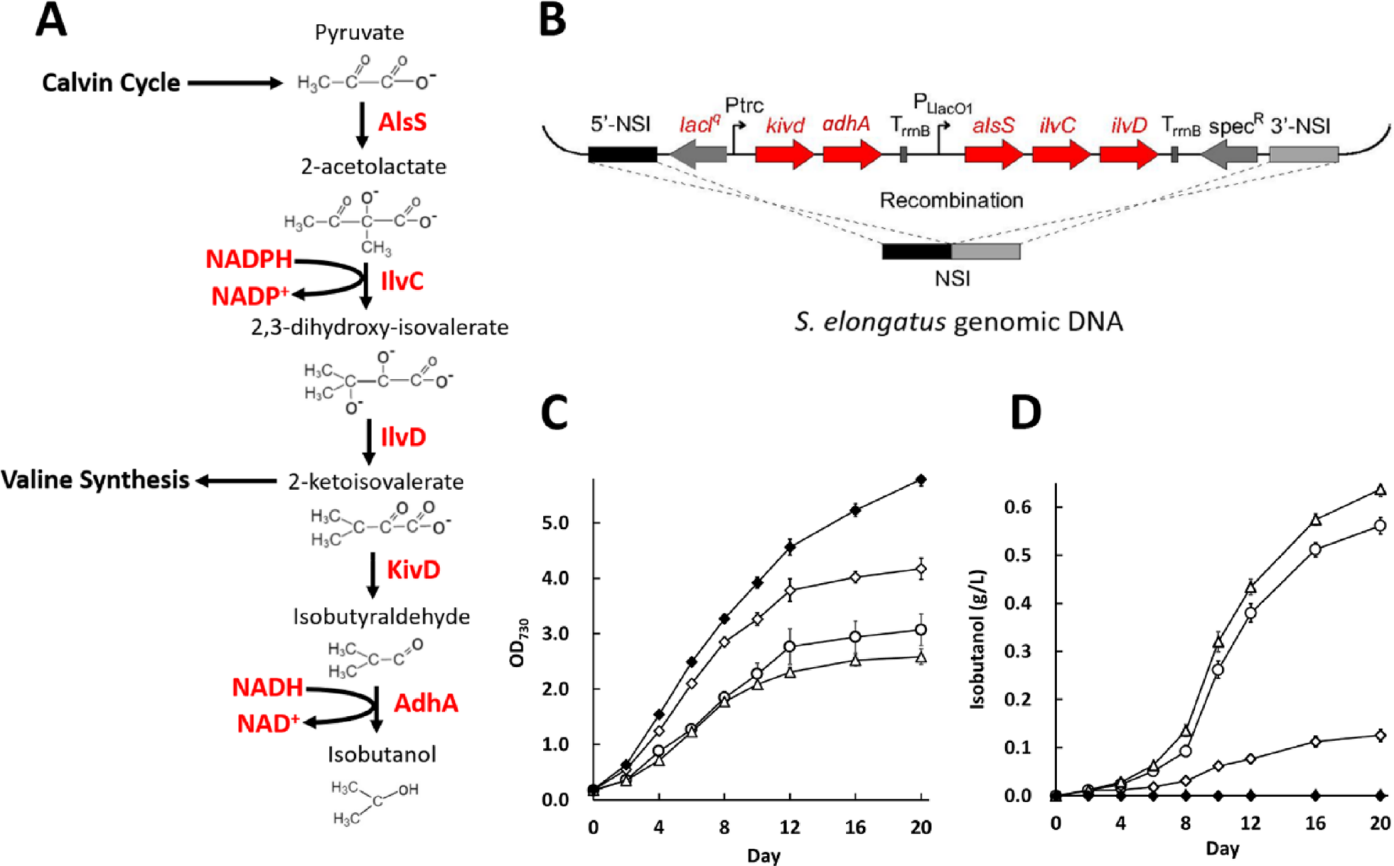
Metabolic engineering of *S. elongatus* to produce isobutanol. **A** Enzymes that catalyze the metabolic pathway from pyruvate to isobutanol. **B** Schematic representation of recombination to integrate *kivd*, *adhA*, *alsS*, *ilvC* and *ilvD* into the *S. elongatus* genome. **C**, **D** Growth curves (**C**) and isobutanol production titers (**D**) of engineered *S. elongatus*. Black diamond: cultivation of JW10 (plasmid pAM2991 integrated in *S. elongatus*) in BG11. White diamond: cultivation of JW11 (plasmid pJW12 integrated in *S. elongatus*) in BG11. Circle: cultivation of JW11 in BG11 with 1.47% NaCl. Up-pointing triangle: cultivation of JW11 in BG11 with 2% sea salt

To investigate how to improve isobutanol production of engineered cyanobacteria using environmental stress, a plasmid pJW12 containing the genes *kivD* and *adhA* from *L. lactis* under the control of the promoter* P*trc, the gene *alsS* from *B. subtilis*, and the genes *ilvC* plus *ilvD* from *E. coli* under the control of the promoter *P*_L_lacO_1_, was constructed (Table 1). The backbone of pJW12 is the plasmid pAM2991 with a neutral site I (NSI), which can be integrated into the *S. elongatus* genomic DNA via homologous recombination (Fig. 1B). The wild-type *S. elongatus* was transformed with pAM2991 and pJW12, resulting in recombinant strains JW10 and JW11, respectively. JW10 produced no observable isobutanol as expected, while JW11 produced approximately 0.126 g/L of extracellular isobutanol within 20 days, which is similar to the unoptimized titers as previously reported [22–24, 33].

**Table 1:** Plasmids and strains used in the study

Name	Genotype	Sources
Plasmids		
pAM2991	NSI targeting vector *P*trc; *Spec*^r^	Addgene #40248
pJW11	*Spec*^r^; NSI targeting; *P*trc::*Kivd*, *adhA*	This study
pJW12	*Spec*^r^; NSI targeting; *P*trc::*Kivd*, *adhA*, *P*_Ll_acO_1_:*alsS*, *ilvC*, *ilvD*	This study

	Relevant genotypes	
Strains		
*S. elongatus*	Wide type	ATCC #33912
JW10	pAM2991 integrated at NSI in *S. elongatus* genome	This study
JW11	pJW12 integrated at NSI in *S. elongatus* genome	This study

The impacts of environmental stress on isobutanol production of engineered *S. elongatus* were then examined. Nitrogen depletion, which has been reported to enhance the lipid accumulation of microalgae, was first investigated. However, a 70% reduction in NaNO_3_ in the BG11 medium apparently inhibited growth of the JW11 strain even though it was inoculated with cultures to maintain the initial OD_730_ of 1.5. Thus, the isobutanol production titer was not enhanced (data not shown). High salinity was introduced by adding 2% sea salt into the BG11 medium. Although the final cell density was only ~ 60% of that without the addition of sea salt (Fig. 1C), the isobutanol titer was elevated fivefold, reaching 0.637 g/L within 20 days (Fig. 1D). Considering the sea salt used comprised of 73.6% of NaCl and 26.4% of other elements, 1.47% NaCl was also added to the medium for JW11 cultivation to confirm that the sea salt-stimulated effect for isobutanol production was attributed to NaCl. The resulting isobutanol titer was similar to when the 2% sea salt was used (Fig. 1D), indicating that osmotic stress was the primary contribution.

All biobutanol production including isobutanol and *n*-butanol through engineering cyanobacteria are listed in Table 2. Biobutanol titers could reach 100–1000 mg/L via introducing essential genes for biobutanol production and further specific modifications either genetically or in culture conditions. Miao et al. recently reported the highest isobutanol titer (911 mg/L) within 40 days through an adjustment of the media pH to neutral during cultivation [24]. Since the cultivation time varies greatly in all reports, likely from different culture conditions, such as initial OD_730_, light intensities, aeration rates and other factors, productivity was compared with reported biobutanol production as titer per cell density (mg/L/OD), instead of as titer per time. Along with Miao et al., we have achieved relatively high productivities (> 200 mg/L/OD) for isobutanol production thus far, which suggests that these modifications in culture conditions are economical and effective approach for enhancing isobutanol production for engineered cyanobacteria compared with modifying the host genotype.

**Table 2:** Comparison of metabolic engineered cyanobacteria for biobutanol production

Product	Host	Engineering genes	Specific modification	Titer productivity	References
Isobutanol	*S. elongatus*	*alsS ilvC ilvD* in NSII *kivD yqhD* in NSI	Initial OD_730_ at 1.0 Enhancing rubisco	450 mg/L 150 mg/L/OD	[11]
Isobutanol	*Synechocystis*	*kivD adhA*	Mixotrophic condition In situ removal of isobutanol	298 mg/L 24.8 mg/L/OD	[23]
Isobutanol	*S. elongatus*	*alsS ilvC ilvD* in NSII *kivD yqhD* in NSI	Deleting a *glgC* gene	550 mg/L 122 mg/L/OD	[22]
Isobutanol	*Synechocystis*	*alsS ilvC ilvD kivD slr1192*	HCl-titrated culture	911 mg/L 227 mg/L/OD	[24]
Isobutanol	*Synechocystis*	*kivD slr1192*	Addition of isobutyraldehyde	290 mg/L 72.5 mg/L/OD	[33]
Isobutanol	*S. elongatus*	*alsS ilvC ilvD kivD adhA* in NSI	High salinity stress	637 mg/L 290 mg/L/OD	This study
*n*-Butanol	*S. elongatus*	*ter* in NSI *atoB adhE2 crt hbd* in NSII	Anoxic condition	14.5 mg/L 5.8 mg/L/OD	[7]
*n*-Butanol	*S. elongatus*	*ter* in NSI *nphT7 bldh yqhD phaJ phaB* in NSII	Engineering ATP consumption	30 mg/L 6.0 mg/L/OD	[8]
*n*-Butanol	*Synechocystis*	*ter phaJ pduP nphT7 fadB slr1192*	Modular pathway engineering	836 mg/L 199 mg/L/OD	[10]
*n*-Butanol	*S. elongatus*	*ter* in NSI *nphT7 pduP yqhD phaJ phaB* in NSII	Using oxygen-tolerant PduP	404 mg/L 66.7 mg/L/OD	[9]

### *Synechococcus elongatus* substantially alters metabolic profiles in response to high salinity stress

**Fig. 2:**
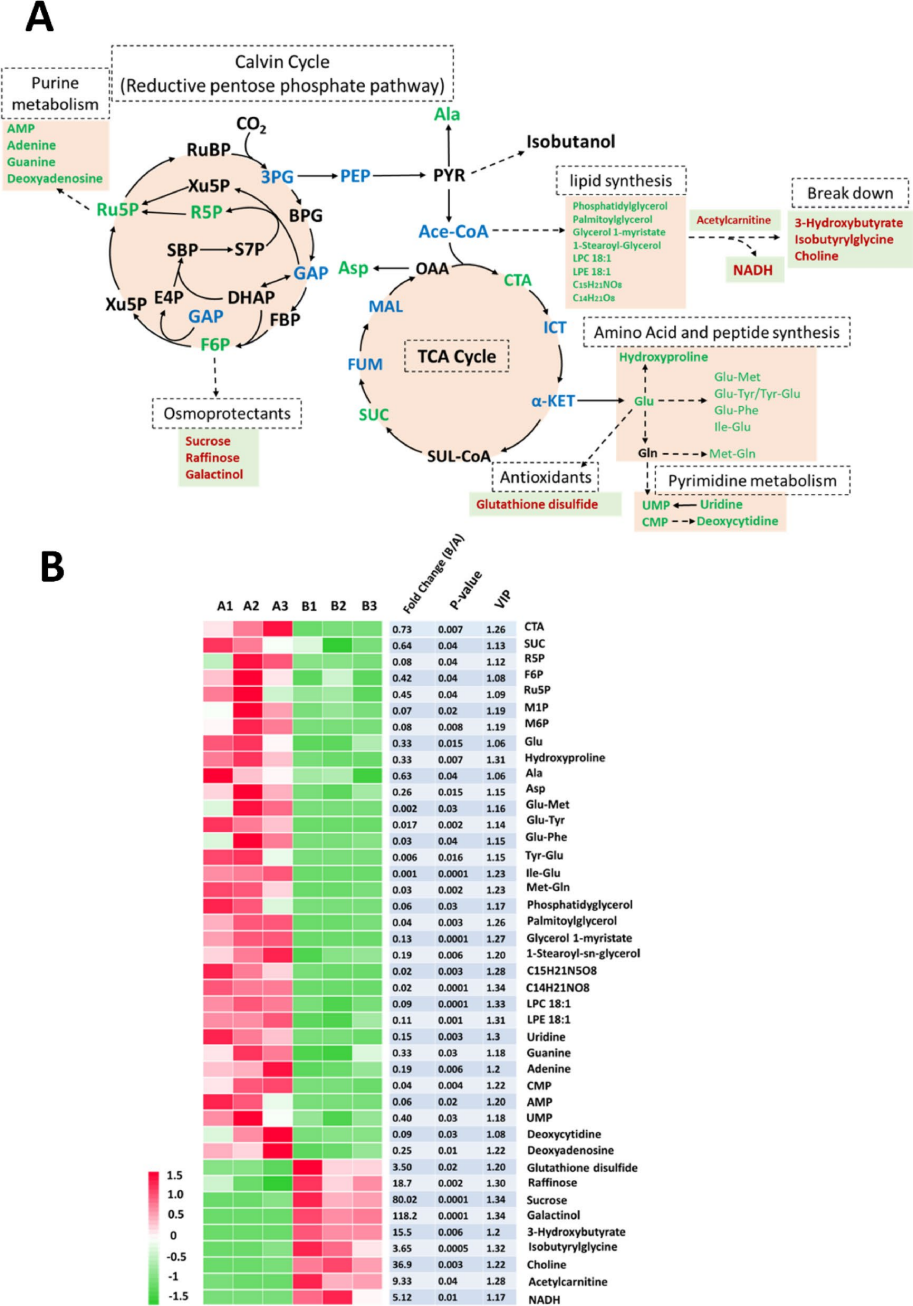
Metabolomics analysis of engineered *S. elongatus* under high salinity condition. **A** Schematic diagram of cyanobacterial central metabolic pathways. Red and green represent up and downregulated metabolites identified from untargeted metabolomics, respectively. Blue represent downregulated metabolites identified from Fig. 3. *Ru5P* ribulose-5-phosphate, *RuBP* ribulose-1,5-bisphosphate, *3-PG* 3-phosphoglycerate, *BPG* 1,3-diphosphoglycarate, *GAP* glyceraldehyde-3-phosphate, *DHAP* dihydroxyacetone phosphate, *F6P* fructose-6-phosphate, *FBP* fructose-1,6-bisphosphate, *E4P* erthrose-4-phosphate, *SBP* sedoheptulose-1,7-bisphosphate, *S7P* sedoheptulose-7-phosphate, *R5P* ribose-5-phosphate, *PEP* phosphoenolpyruvate, *PYR* pyruvate, *Ace-COA* acetyl-CoA, *OAA* oxaloacetate, *CTA* citrate, *ICT* isocitrate, *α-KET* α-ketoglutarate,* SUL-CoA* succinyl-CoA, *SUC* succinate, *FUM* fumarate, *MAL* malate, *Ala* alanine, *Asp* aspartate, *Glu* glutamate, *Gln* glutamine, *Met* methionine, *Tyr* tyrosine, *Phe* phenylalanine, *Ile* isoleucine. **B** Heatmap and statistical analysis of 42 differential accumulated metabolites localized in cyanobacterial central metabolic pathway

To explore why osmotic stress stimulated isobutanol production, an untargeted metabolomics analysis for the JW11 strain was performed under normal and high-salinity conditions. A total of 105 metabolites were annotated and quantified based on the LC–ESI–MS/MS spectra (Additional file 1: Table S2). Metabolites with univariate statistical significance (fold change > 1.4 and *p* < 0.05) were selected for further analysis. A total of 21 and 32 differentially accumulated metabolites were identified between the control and the stressed cells in the ESI^+^ and ESI^−^ modes, respectively. A total of 42 differentially accumulated metabolites were functionally localized in the central metabolic pathway of the cyanobacteria, as shown in Fig. 2A.

Among the 42 critical metabolites, R5P, F6P, and Ru5P were identified from the Calvin cycle, through which cyanobacteria are employed for carbon assimilation, and these were downregulated 12.5-, 2.4-, and 2.2-fold, respectively, under osmotic stress. Citrate and succinate from the TCA cycle, which are required for aerobic respiration, were downregulated 1.4- and 1.6-fold, respectively. A weakening of the Calvin cycle and TCA cycle explained cyanobacterial growth inhibition under the high salinity condition. Biosynthesis metabolisms were also attenuated due to osmotic stress. For example, four amino acids were identified and downregulated at least 1.6-fold under the high salinity condition. Six dipeptides showed dramatic reductions, indicating that protein synthesis was also inhibited. Moreover, purine and pyrimidine metabolism, responsible for nucleic acid synthesis and derived from R5P and glutamate, respectively, were strongly suppressed under osmotic stress. With respect to lipid metabolism, phosphatidylglycerol, a major component of cellular membrane, and a couple of energy storages, such as palmitoylglycerol, were downregulated more than fivefold under osmotic stress. In contrast, the abundance of lipid-decomposed products, such as 3-hydroxybutyrate and isobutyrylglycine, increased 15.5- and 3.65-fold, respectively. The abundance of acetylcarnitine, which is a lipid transporter for aerobic respiration, also reasonably increased 9.33-fold under the high-salinity condition, confirming the acceleration of lipid decomposition. Interestingly, the abundance of NADH increased 5.1-fold under osmotic stress (Fig. 2). It is unlikely that the TCA cycle contributed to the additional NADH, since it is inhibited under osmotic stress. The enhanced lipid decomposition, which is also a pool for NADH in vivo, probably led to the increased abundance of NADH. It is reasonable to speculate that cyanobacteria utilized lipid decomposition to provide a sufficient amount of NADH for aerobic respiration due to inhibition of the TCA cycle. It is also worth mentioning that one of the exogenous enzymes that was introduced for isobutanol biosynthesis, AdhA, is a NADH-dependent enzyme [13]. Therefore, we speculate that the elevated NADH abundance was likely one of the reasons for the isobutanol production enhancement under osmotic stress. In addition, some osmo-protectants, such as sucrose, raffinose and galactinol, were upregulated 20–100-fold under salt stress, consistent with previous reports [34]. An oxidized form of the antioxidant glutathione disulfide was upregulated 3.5-fold, indicating that the osmotic-stressed cells were suffering from oxidative damage.

**Fig. 3:**
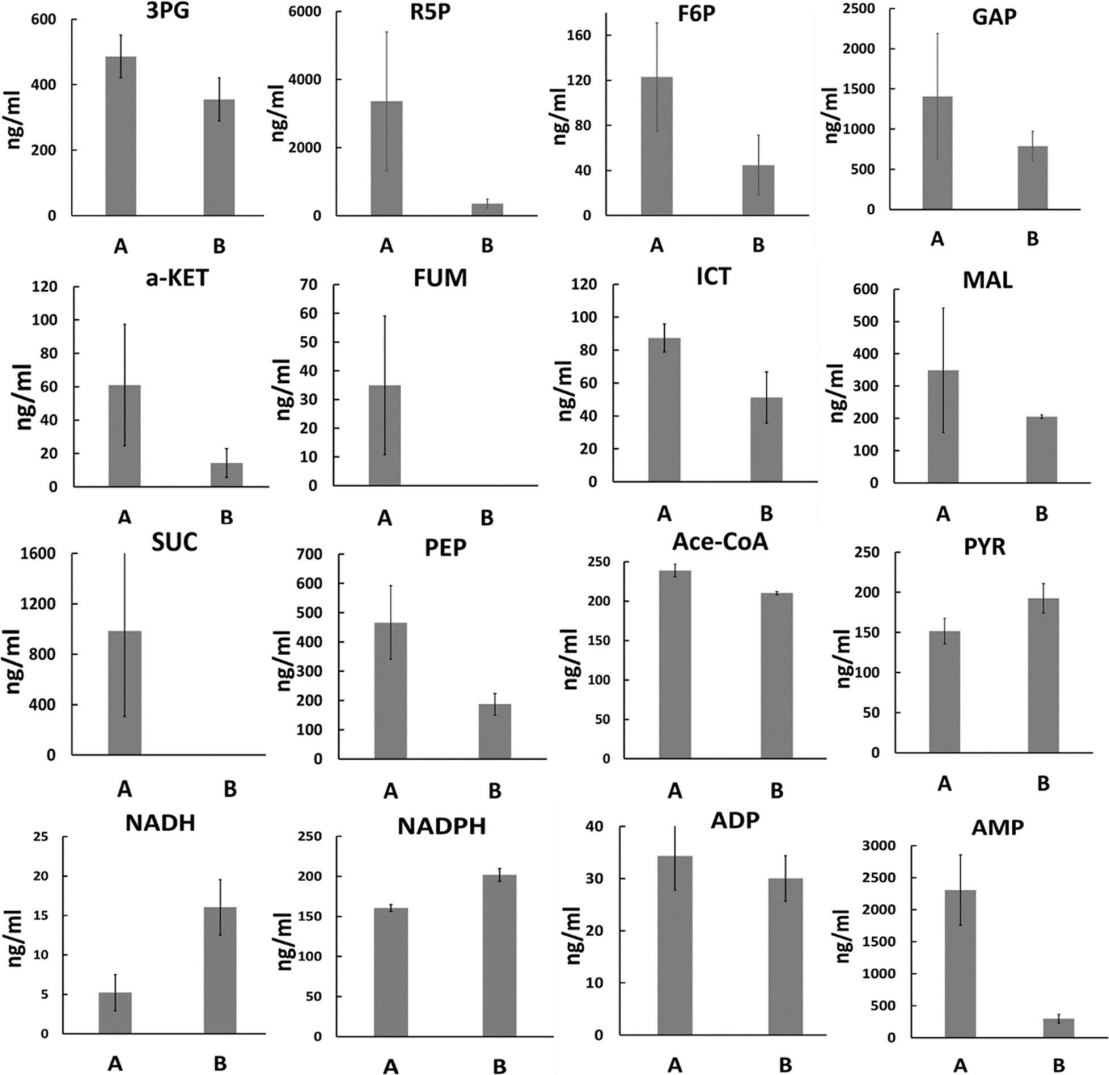
In vivo concentration comparison of energy metabolites under normal (Group A) and high salinity conditions (Group B). Abbreviations of energy metabolites are the same with that of in Fig. 2. Absence of data for FUM and SUC does not mean they were non-existent in vivo, but means the detected amounts were below the minimal concentrations of standards, so the absolute concentrations were not available

To verify the changes of metabolic profiles under high salinity stress, absolute quantification assays were performed for selected energy metabolites via LC–MS using known concentrations of chemicals as standards. Consistent with the untargeted metabolomics profiles, 3PG, R5P, F6P, and GAP, which are involved in the Calvin cycle, and α-ketoglutarate, fumarate, malarate, isocitrate, and succinate, involved in the TCA cycle, were all downregulated under high-salinity condition. The metabolites connecting the Calvin and TCA cycles, such as PEP and acetyl-CoA, were also downregulated, whereas the isobutanol precursor pyruvate did not decrease under high salinity condition (Fig. 3). Although the key energy metabolite ATP could not be detected, ADP and AMP, the two precursors of ATP, were downregulated under high-salinity condition. Given that the growth of cyanobacteria and TCA cycle were inhibited, ATP was also assumed to be downregulated under high-salinity condition. NADH was upregulated threefold, consistent with the untargeted metabolomics result, while another redox equivalent NADPH was upregulated 1.3-fold (Fig. 3). Notably, among the five exogenous enzymes introduced for isobutanol biosynthesis, AdhA is a NADH-dependent enzyme, but IlvC is a NADPH-dependent enzyme [35]. A question now arises as to whether the increased abundance of NADH itself or the increased abundances of both redox equivalents contributed to the enhanced isobutanol production.

### High salinity stimulates isobutanol production by decomposing lipid to accumulate NADH and by increasing membrane permeability

**Fig. 4:**
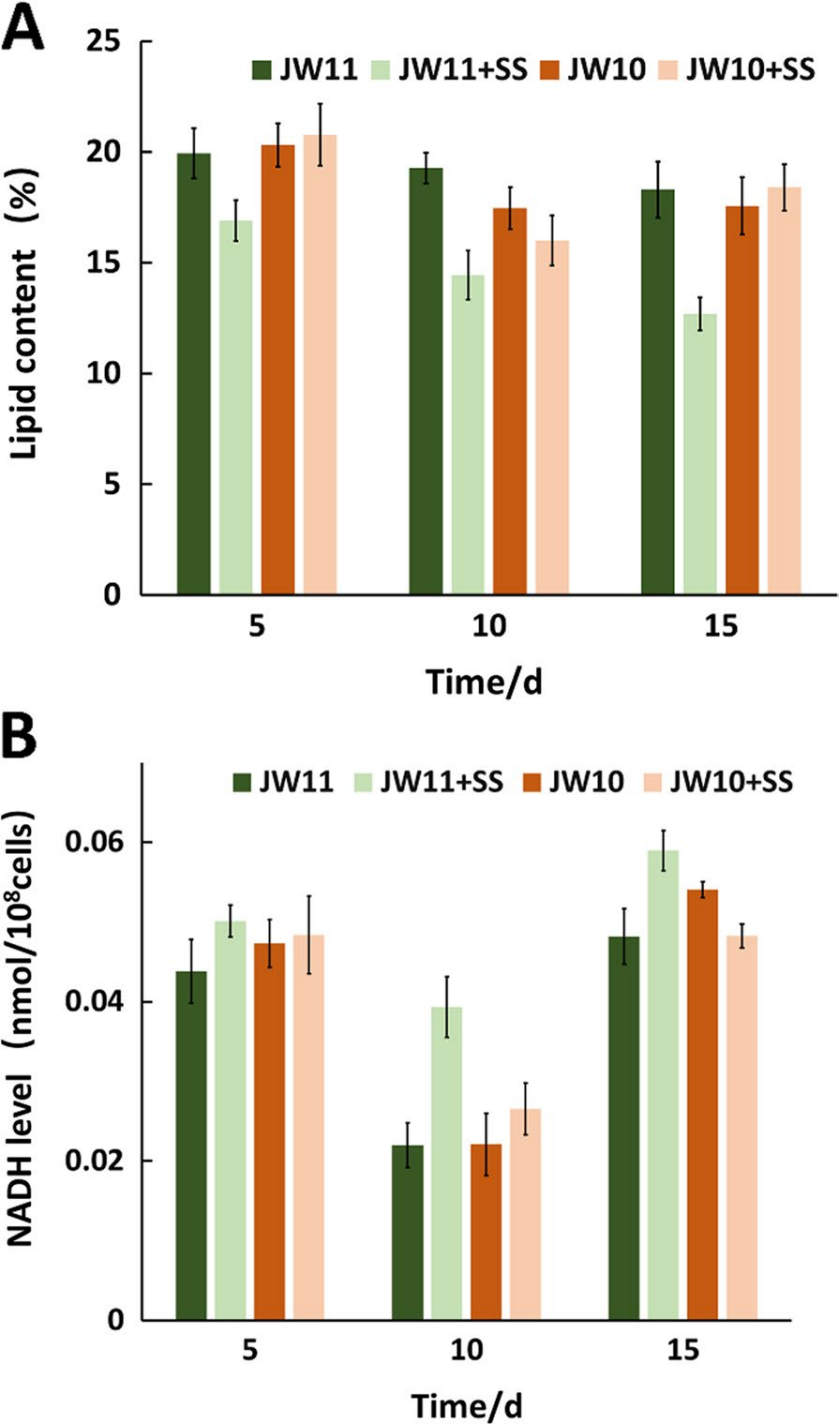
Analysis of lipid contents and NADH levels of engineered *S. elongatus* under normal and high salinity stress conditions cultured at days 5, 10 and 15. **A** Lipid contents. **B** NADH levels

Analysis for untargeted metabolomics and energy metabolites undoubtedly demonstrated that TCA cycle was inhibited, which is the major NADH pool, but lipid decomposition was upregulated under high salinity stress, which is another potential NADH pool. We, therefore, quantified the lipid contents and NADH levels at different culture stages of the isobutanol-producing JW11 strain and the control JW10 strain under normal and high salinity stress conditions. For the JW11 strain, the lipid contents dropped by 3.0%, 4.85% and 4.02%, while the NADH level increased 1.14-, 1.79- and 1.23-fold under the high salinity stress at culture days 5, 10 and 15, respectively. For the JW 10 strain, the reduction of lipid contents in proportion to increases in NADH levels under high salinity stress was not observed (Fig. 4). These results clearly indicate that the upregulated NADH was due to acceleration in lipid decomposition in the JW11 strain, in which extra NADH is required for the isobutanol-producing pathway. This conclusion was also supported by quantifying total free fatty acid contents of the JW11 and JW10 strains under normal and high salinity stress conditions (Additional file 1: Fig. S2).

We then quantified abundances of another redox equivalent NADPH for the JW11 and JW10 strains under normal and high salinity stress conditions. Although the level of NADPH was upregulated 1.2-fold at day 10 under high salinity stress, which was consistent with the result in Fig. 3, the levels were downregulated 1.6- and 1.4-fold under high salinity stress at days 5 and 15, respectively (Additional file 1: Fig. S1). In autotrophic cyanobacteria, the major pool for NADPH is the light-driven electron transport chain. The generated NADPH then serves as a reducing driver for biosynthetic and antioxidant systems. Untargeted metabolomics suggest that the biosynthetic metabolism was inhibited, whereas antioxidative activity appeared to be enhanced under high salinity stress as determined by the quantification of reactive oxygen species (ROS) and Glutathione (GSH) (Additional file 1: Fig. S1). The flexible utilization and regeneration systems for NADPH may be the reason for the fluctuations of its abundance. We, therefore, excluded the possibility that the level of NADPH impacted the isobutanol production in the JW11 strain.

**Fig. 5:**
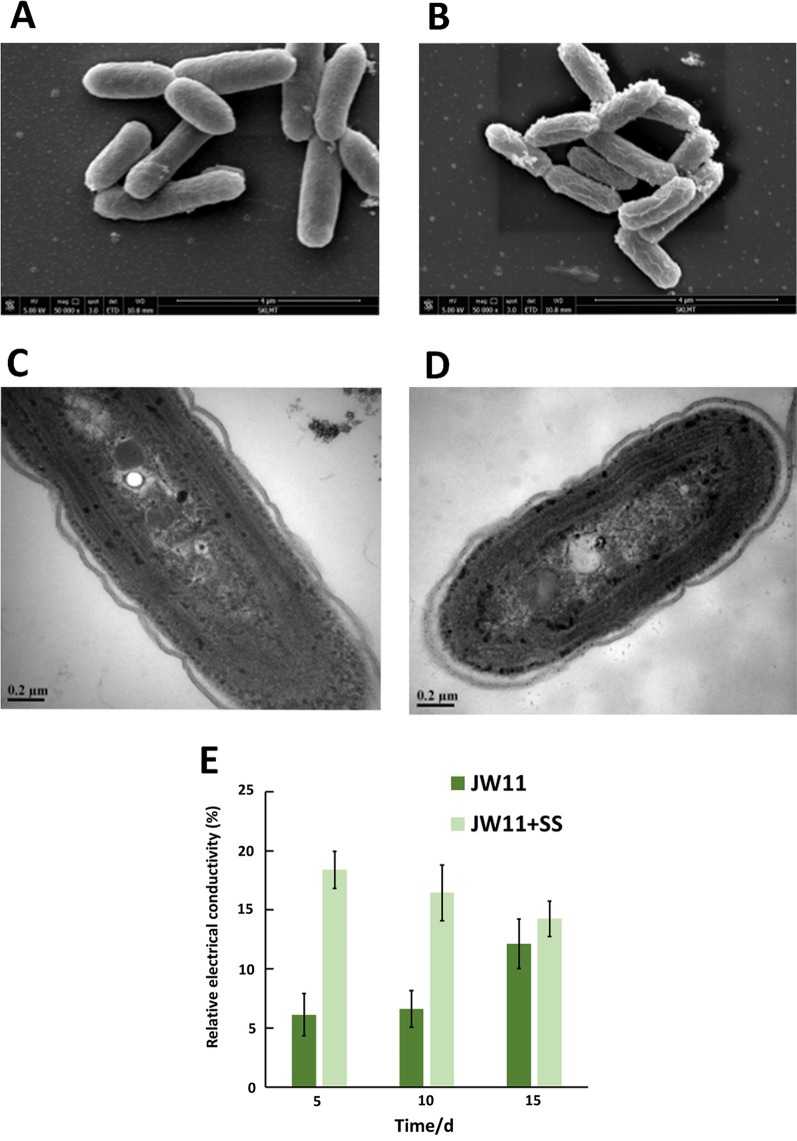
Morphological observation and membrane permeability of engineered *S. elongatus*. SEM images for the stress-free cells (**A**) and salinity stressed cells (**B**). TEM images for the stress-free cells (**C**) and salinity stressed cells (**D**). Relative electrical conductivity (**E**)

To gain more insights into how osmotic stress enhanced isobutanol production of the engineered *S. elongatus*, we analyzed cell morphology and membrane permeability under normal and high salinity conditions. Scanning electron microscopy (SEM) results showed that the osmotic-stressed cells became notably wrinkled and slimmer with a rod-shaped cell’s diameter of 0.61 ± 0.04 μm, compared to a stress-free cell’s diameter of 0.81 ± 0.07 μm (Fig. 5A, B). Since cell morphology is partially influenced by membrane layers that encase the microalgal cells, we characterized the membrane permeability by quantifying the relative conductivity of membranes as previously reported [36]. Figure 5E shows that the relative conductivities under salinity stress were threefold higher than that of no salt added in the first 10 days of cultivation, indicating that the membrane permeability increased significantly. Transmission electron microscopy (TEM) results provided more evidence for showing that the cell’ membrane was partially disturbed by osmotic stress (Fig. 5C, D). The increased membrane permeability was likely another reason for the enhanced production titer as it facilitated leakage of isobutanol, thereby reducing toxicity to cyanobacterial cells.

### A novel biotechnology for mixing synthetic wastewater with seawater to produce isobutanol

Nitrogen and phosphorus elements are considered to be the most critical nutrient factors that affect the growth and metabolism of aquatic cyanobacteria [37]. Wastewater typically contains different levels of nitrogen and phosphorus elements, which may provide necessary nutrients for cyanobacterial growth. In addition, seawater can provide the high salinity condition required for the massive production of isobutanol using engineered *S. elongatus*. Consequently, the recent proposed biotechnology of mixing wastewater with seawater to produce bioenergy by microalgae offers an attractive way to cultivate the engineered *S. elongatus* for sustainable isobutanol production [38].

**Fig. 6:**
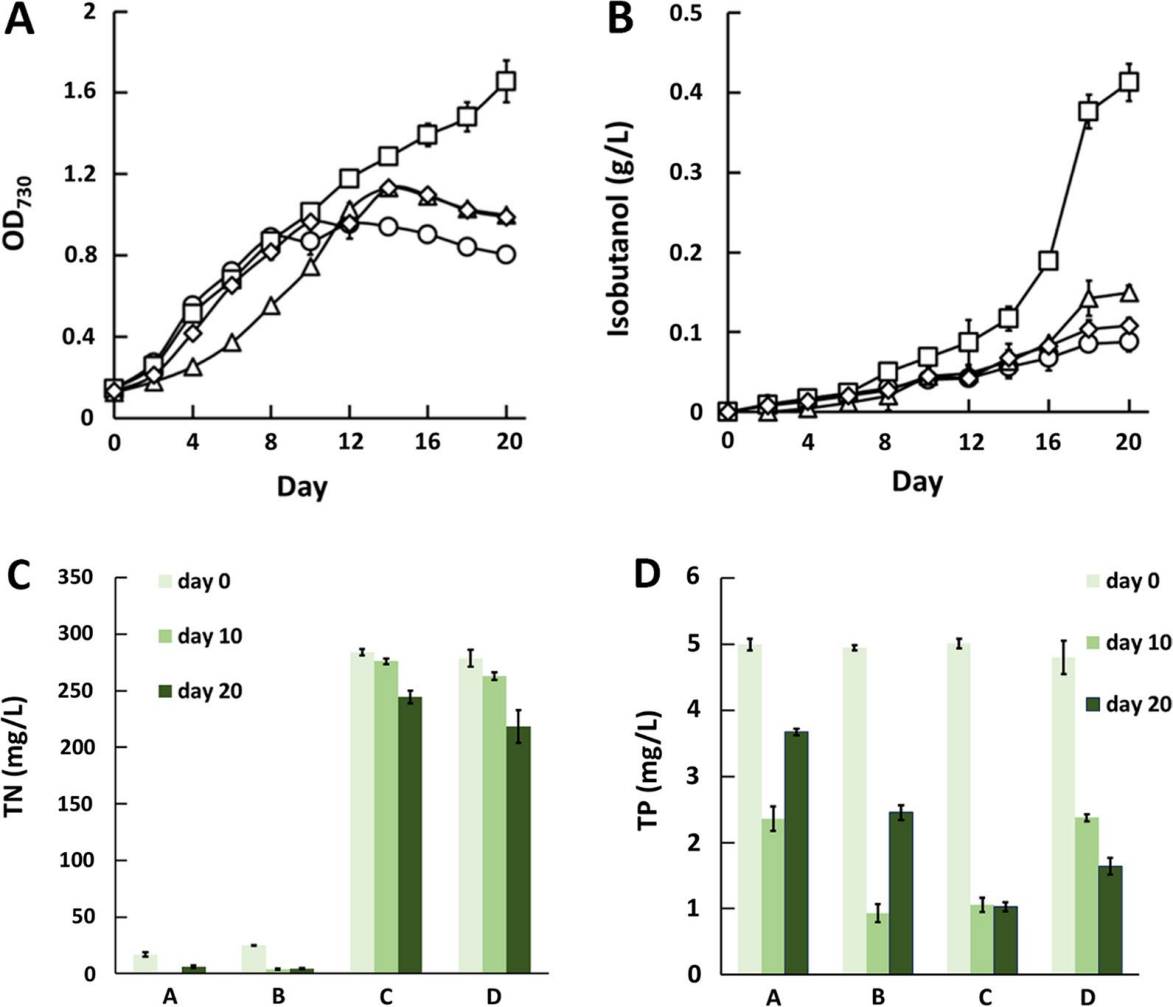
Cultivation of engineered *S. elongatus* to produce isobutanol through a system by mixing synthetic wastewater with seawater. **A** Growth curves and **B** isobutanol production titers under different nitrogen types and concentrations. Circle: basic-NH_4_^+^. Diamond: basic-NO_3_^−^_._ Up-pointing triangle: high-NH_4_^+^. Square: high-NO_3_^−^. **C** Total nitrogen and **D** phosphorous consumptions under different nitrogen types and concentrations. **A** Basic-NH_4_^+^. **B** Basic-NO_3_^−^_._ **C** High-NH_4_^+^. **D** High-NO_3_^−^

First, artificial wastewater was synthesized as previously reported [39], containing all of the necessary elements for cyanobacterial growth. However, the synthetic wastewater was composed of either 17 mg/L of NH_4_^+^–N or 17 mg/L of NO_3_^−^–N designated as basic-NH_4_^+^ and basic-NO_3_^−^, respectively, far below the 247 mg/L of NO_3_^−^–N in the BG11 medium. Therefore, NH_4_^+^–N or NO_3_^−^–N sources were added to the wastewater, reaching final nitrogen concentrations equal to that of the BG11 medium, designated as high-NH_4_^+^ and high-NO_3_^−^, respectively. The phosphorus content in the basic synthetic wastewater was similar to that of the BG11. Artificial seawater was also synthesized to contain 3.5% salt salinity. To maintain an optimal salinity of 2% for isobutanol enhancement, the seawater and wastewater were mixed at a ratio of 1:0.75. The results showed that the wastewater of the basic-NH_4_^+^ and basic-NO_3_^−^ could support the growth of the engineered *S. elongatus* until days 8 and 10, respectively, and that both could produce isobutanol with maximal titers of ~ 0.1 g/L at day 20. The addition of NH_4_^+^–N to the basic-NH_4_^+^ significantly inhibited growth from day 0 to day 12, but produced 0.15 g/L of isobutanol at day 20. The addition of NO_3_^−^–N to the basic-NO_3_^−^ supported growth of the engineered *S. elongatus* continuously, producing 0.413 g/L of isobutanol, which was similar to that of BG11 plus 2% sea salt (Fig. 6A, B). Nitrogen and phosphorus utilization were also measured for all cultivations. As expected, the total nitrogen was nearly exhausted for the basic-NH_4_^+^ and basic-NO_3_^−^ at day 10. Due to the addition of excessive NH_4_^+^–N and NO_3_^−^–N, 20–60 mg/L of total nitrogen was consumed by the engineered *S. elongatus*, while phosphorus utilizations were enhanced at least twofold (Fig. 6C, D).

These results indicate that the basic synthetic wastewater containing 17 mg/L of NH_4_^+^–N or NO_3_^−^–N can support engineered *S. elongatus* to produce isobutanol. In general, wastewater of NH_4_^+^–N, such as municipal and farming wastewater, contains at least 20 mg/L of nitrogen, which meets the requirements for cultivating engineered *S. elongates* [40]*.* Nevertheless, these results demonstrate that NO_3_^−^–N is the most crucial factor for affecting the growth and isobutanol production of engineered *S. elongatus*. Wastewater containing high-strength NO_3_^−^–N is being produced by a variety of industries, such as for fertilizers, explosives, and metal finishing [41, 42]. For example, the nitrate concentrations in stainless manufacturing and the fertilizer industry are normally in the range of 0.7–1.0 g/L, which can sufficiently support engineered *S. elongatus* to grow and produce isobutanol [43]. High concentrations of NH_4_^+^–N apparently inhibit cyanobacterial growth, as reported previously for other microalgal species. The molecular basis for NH_4_^+^–N inhibition of algal photosynthesis remains unclear. The best evidence proposes NH_4_^+^ uncoupling of the electron transport in photosystem II by the breakdown of proton gradients necessary to drive photophosphorylation or inhibition via NH_4_^+^ competition with H_2_O during oxidation reactions, leading to O_2_ evolution, or both [44, 45].

## Conclusions

In summary, our study developed a novel, third-generation biorefinery process to produce isobutanol by converting inorganic CO_2_. We first discovered that high salinity stress could significantly enhance isobutanol production of engineered *S. elongatus*, then multi-perspective analysis revealed that increasing NADH and membrane permeability under salinity stress contributed to the enhancement of isobutanol production. We then demonstrated that the biotechnology for mixing wastewater with seawater can be employed for cultivating engineered *S. elongatus*. NO_3_^−^–N was the most important factor impacting growth and isobutanol production. Taken together, the findings from this study are of great significance to biofuel production and resource recycling.

## Methods

### Stains and culture conditions

*Synechococcus elongatus* was purchased from the American Type Culture Collection and cultivated at 30 °C with a constant light of 50 μmol photon m^−2^ s^−1^. It was then shaken at 150 rpm in a BG11 medium containing 40 mM NaHCO_3,_ as previously reported [11]. A total of 20 μg/mL of Spectinomycin, 0.1 mM of isopropyl beta-d-thiogalactopyranoside (IPTG), and 2% sea salt (Sigma-Aldrich) were added as indicated. A total of 2 mL of the culture was sampled from screw-capped flasks every 2 days and measured for cell growth and isobutanol quantification. In addition, 2 mL of fresh BG11 medium with the indicated components was then added back to the flask. *Escherichia coli* DH5α (Invitrogen) was used for cloning, and it was cultivated under 37 °C and shaken at 200 rpm in Luria–Bertani media.

### Plasmid construction and transformation of cyanobacteria

The plasmids constructed in this work are listed in Table 1, and briefly described below. The primers are listed in Additional file 1: Table S1. The plasmid pAM2991 was used as a targeting vector as it has an NSI that can be integrated into the *S. elongatus* genomic DNA. *KivD* (LLKF_1386) *and adhA* (LLKF_1981) were amplified from the genome of *Lactococcus lactis* (NC_013656) using primers LJW105, LJW106, LJW107, and LJW108, respectively, and inserted into EcoRV and NheI sites of pAM2991 under an IPTG-inducible promoter* P*trc, resulting in the plasmid pJW11. A promoter, *P*_L_lacO_1,_ was synthesized by the Beijing Genomics Institut (BGI). *AlsS* (BSU_36010) was amplified from the genome of *B. subtilis* 168 (NC_000964.3) using primers LJW109 and LJW110. *IlvC* (ECK_3766) and *ilvD* (ECK_3763) were amplified from the *E. coli* MG1655 genome (NC_000964.3) using primers LJW111, LJW112, LJW113, and LJW114, respectively. *P*_L_lacO_1_, *alsS*, *ilvC*, and *ilvD* fragments were inserted into the plasmid pJW11 between the Stu1and BamH1 sites, resulting in the plasmid pJW12. All of the cloning experiments were completed by Gibson Assembly or In-Fusion cloning.

Transformation of *S. elongatus* was conducted as previously described [11]. Cyanobacterial transformants with the targeting vectors were selected on BG-11 agar plates supplemented with antibiotics as appropriate. Recombinants were confirmed using PCR and sequencing. The strains used and constructed are listed in Table 1. Briefly, strain JW10 was constructed by the homologous recombination of pAM2991 at NSI of the wild-type *S. elongatus*. Strain JW11 was constructed by the homologous recombination of pJW12 at the NSI of the wild-type *S. elongatus*.

### Analytical techniques of cell growth, isobutanol production, NADH, NADPH, lipid contents, membrane permeability and antioxidative ability

Cell growth was monitored using an optical density of 730 nm via a spectrophotometer (UNICO). The isobutanol was measured and quantified using gas chromatography (Shimadzu GC2014). Specifically, 2 mL of cyanobacterial culture was centrifuged at 5000×*g* for 10 min, and the supernatant was filtered through a 0.22-um filter. The purified samples were then applied to a GC equipped with a column (30 m × 0.32 mm × 0.25 μm, Agilent Technologies). Nitrogen was the carrier gas with a 63.4-mL/min flow rate. Methanol was used as an internal standard. At least three replicates of all assays were performed.

Intracellular abundances of NADH and NADPH were determined using NADH Assay Kit with WST-8 (Beyotime Biotech, Shanghai, China) and NADPH Assay Kit with WST-8 (Beyotime Biotech, Shanghai, China) according to the manufacturer’s instructions. The concentrations of NADH and NADPH were calculated according to the NADH or NADPH standard curves.

Lipid contents were determined based on a previously described method [36]. Briefly, 100 mL of cyanobacterial cells were harvested and dried before weighing (*m*_1_). The dried powder was transferred into 10 mL of a chloroform/methanol (2:1, v/v) mixture, ultrasonicated and centrifugated at 4000×*g* for 10 min. The extraction process was repeated twice. All supernatants were collected in a separated funnel, and 4 mL of sodium chloride solution (0.9%) was added. The extract was then mixed and allowed to undergo phase separation for 15 min, and the lower organic phase was collected with a glass tube. The lower phase containing extracted total lipids was blow-dried with nitrogen in a water bath at 60 °C, and dried to constant weight (*m*_2_) in an oven. Lipids contents (%) = *m*_2_/*m*_1_ × 100%.

Relative electrical conductivity was determined based a previously reported method [36]. Briefly, 10 mL of cyanobacterial culture was collected and centrifuged at 5000×*g* for 15 min, supernatant was discarded and the pellets were washed three times with distilled water. The pellets were then resuspended with 5 mL of distilled water before measuring the initial electrical conductivity *R*_0_. After waiting for 30 min, the electrical conductivity *R*_1_ was measured. Then the cyanobacterial mixture was heated in a boiling water bath for 15 min and cooled in the air to room temperature before the conductivity *R*_2_ was measured. The relative electrical conductivity was determined by the following equation:

$${\text{Relative}}\;{\text{electrical}}\;{\text{conductivity}}\left( \% \right) = {{(R_{1} - R_{0} )} \mathord{\left/ {\vphantom {{(R_{1} - R_{0} )} {(R_{2} - R_{0} )}}} \right. \kern-\nulldelimiterspace} {(R_{2} - R_{0} )}} \times 100\% .$$

DCFH-DA fluorescent probes were used to detect intracellular ROS. 10 mL of cyanobacterial culture was collected and centrifuged at 5000×*g* for 5 min. The supernatant was discarded, and the residues were suspended with phosphate buffer saline (PBS). The cell density was controlled at about 1 × 10^8^ cells/mL. DCFH-DA was added at a working concentration of 10 μM, and blowing and mixing were repeated. The cells were incubated at 37 °C for 30–60 min, and centrifuged at 5000×*g* for 5 min to collect the cells. The cells were washed twice with PBS and resuspend to the original volume. Fluorescence detection was performed at 485 ± 20 nm excitation wavelength and 516 ± 20 nm emission wavelength by a fluorescence microplate reader (Varioskan Lux, Thermo Fisher, USA).

Commercially available assay kits (Jiancheng Bioengineering Institute, China) were used to determine the concentrations of GSH. 5 mL of cyanobacterial culture was collected, washed with PBS, and centrifuged at 5000×*g* for 10 min. Next, the supernatant was discarded and 0.5 mL of PBS was added to resuspend the cells. The samples were frozen and thawed repeatedly in liquid nitrogen three times. 0.1 mL of the crushed cell suspension was added to 0.1 mL of precipitator, mixed and centrifuged at 10,000×*g* for 5 min. Finally, the supernatant was ready for GSH detection.

### Metabolomics analysis

Metabolomics profiling was performed using a liquid chromatography electrospray ionization tandem mass spectrometry (LC–ESI–MS/MS) analysis. Specifically, 2 mL of cyanobacterial cells were suspended in 500 μL of a cold mixture (acetonitrile: methanol: H_2_O, 2:2:1, v/v/v). After 1 h of incubation at − 20 °C, the sample was centrifuged at 14,000×*g* for 20 min at 4 °C. The supernatant was collected and dried in a vacuum desiccator. The pellet was treated using 100 μL of an acetonitrile: water mixture (1:1), and the sample was vortexed for 1 min. After further centrifugation at 14,000×*g* for 20 min, 2 μL of the supernatant was analyzed using LC–ESI–MS/MS. High performance liquid chromatography (HPLC) separation was performed using the Agilent 1290 Infinity LC system equipped with an HILIC column. The mobile phase was composed of a solution containing 25 mM of formic acid and 25 mM of aqueous ammonia in water, as well as a B solution containing 100% acetonitrile. The following gradient was established: 95% B solution for 0–1 min, 95–65% B solution for 1–14 min, 65–45% B solution for 14–16 min, 40% B solution for 18–18.1 min, and 40–95% B solution for 18.1–23 min. The samples were kept in an autosampler at 4 °C throughout the analysis.

For the untargeted metabolomics analysis, the metabolites were injected into a time of flight (TOF) 6600 mass spectrometer and detected using the ESI positive and negative ion modes. The temperature of the MS quadrupole and ion source was set at 600 °C. The collision energy was 35 eV. The TOF MS scanning was performed at a range of 60–1000 *m*/*z* with an accumulation time of 0.2 s per spectra. The product ion scan was performed for a mass range of 25–1000 *m*/*z* with an accumulation time of 0.05 s per spectra. Quality control samples were inserted at regular intervals throughout the entire analysis. Metabolite annotation was performed by retrieving the obtained retention indices and mass spectral data from the databases of METLIN and MassBank.

For the targeted energy metabolomics analysis, the metabolites were injected into a QTRAP 5500 mass spectrometer and detected using the ESI negative ion mode. The ESI source conditions were as follows: ion source gas 1: 45, ion source gas 2: 45, curtain gas: 30, source temperature 450 °C, and ion spray voltage floating at − 4.5 kV. Metabolites of interest were identified by comparisons with retention times of standard chemicals ranging from 20 to 5000 ng/μL. All of the ion pair information for the selected energy metabolites are shown in Additional file 1: Table S3.

### Microscopy analysis

For TEM, 1 mL of cyanobacterial culture was taken, centrifuged, and the cyanobacterial precipitation was collected. The cyanobacteria was then washed with PBS. A 2.5% glutaraldehyde solution precooled at 4 °C was slowly added to fix the sample, and stored in a refrigerator at 4 °C for 12 h. Then the solution was discarded, and the sample was washed with PBS three times, fixed with 1% osmic acid solution for 1–2 h and washed with PBS three times before dehydration by a graded series of ethanol (30%, 50%, 70%, 80%, 90%, 95% and 100%) for about 15–20 min at each step, then transferred to absolute acetone for 20 min. Next, the specimen was placed in a 1:1 mixture of absolute acetone and the final Spurr resin mixture for 1 h at room temperature, then transferred to a 1:3 mixture of absolute acetone and the final resin mixture for 3 h and to final Spurr resin mixture for overnight. The specimen was placed in a tube containing Spurr resin and heated at 70 °C for more than 9 h. The specimen was sectioned with a LEICA EM UC7 ultratome and the sections were stained by uranyl acetate and alkaline lead citrate for 5–10 min, respectively, and observed by a Hitachi Model H-7650 TEM.

For SEM, 1 mL of cyanobacterial culture was centrifuged at 5000×*g* for 15 min and the cells were washed with PBS. The supernatant was discarded, 2.5% glutaraldehyde was added, and the cells were fixed at 4 °C for 12 h before washing three times with PBS. Then the supernatant was discarded, and the samples were dehydrated with 30%, 50%, 70%, 80%, 90% and 100% ethanol aqueous solutions sequentially. Samples were centrifuged for 15 min at each step, and then dried in a critical point dryer. After fully drying, the samples were gold-plated, and the morphology and size of cells were observed using a Field emission SEM (FEI Quanta 250 FEG, Thermo, USA).

### Cultivation of engineered *S. elongatus* in artificial wastewater mixed with seawater

The seawater and wastewater used in this study were synthesized artificially. The composition of the synthetic wastewater was the same as that previously described [39]. NH_4_^+^–N and NO_3_^−^–N were added as indicated in the experiments. The artificial seawater was prepared from sea salt with a salinity of 3.5%. The seawater and wastewater were mixed at a ratio of 1:0.75, which resulted in a final salinity of 2% in the medium. A total of 2 mL of culture was sampled from the flask every 2 days and measured for cell growth and isobutanol quantifications. A total of 10 mL of culture was sampled from the flask every 10 days and measured for total nitrogen and phosphorus quantifications. The total nitrogen content was determined using a total nitrogen analyzer (LH-3BN, China). The total phosphorus was determined using a multi-parameter water quality analyzer (LH5B-3B, China). At least three replicates of all the assays were performed. The synthetic wastewater used in this study included (in mg L^−1^): glucose: 220, sodium acetate: 220, NH_4_Cl: 149, KH_2_PO_4_: 48.6, NaHCO_3_: 286, MgSO_4_·7H_2_O: 50, and CaCl_2_: 4. A total of 1 mL of a trace element solution was added to the simulated wastewater. The composition of the trace element solution was as follows: (in mg L^−1^): AlCl_3_: 90, CuSO_4_·5H_2_O: 50, MnCl_2_·4H_2_O: 120, ZnSO_4_·7H_2_O: 120, H_3_BO_3_: 150, NiCl_2_·6H_2_O: 90, NaMoO_4_·2H_2_O: 60, and CoCl_2_·7H_2_O: 150.

## Supplementary Information

**Additional file 1: Table S1.** Primers sequences. **Table S2.** Identification of metabolites from metabolomics between JW11 strain (A) and JW11 strain cultured with 2% sea salt (B). **Table S3.** Targeted energy metabolites from LC–MS between JW11 strain (A) and JW11 strain cultured with 2% sea salt (B). **Figure S1.** Analysis of NADPH level and antioxidative ability of engineered *S. elongatus* under normal and high salinity stress conditions cultured at days 5, 10 and 15. (A) NADPH level. (B) reactive oxygen species level. (C) Glutathione level. (D) Schematic representation of antioxidative stress system in vivo. **Figure S2.** Analysis of total fatty acid of engineered *S. elongatus* under normal and high salinity stress conditions cultured at days 5, 10 and 15.

## Data Availability

All the data analyzed during this study have been included in this article.
