# Supplementary material for: Establishment of a resource recycling strategy by optimizing isobutanol production in engineered cyanobacteria using high salinity stress
Source: Biotechnol Biofuels. 2021 Aug 30;14:174. doi: 10.1186/s13068-021-02023-8 (PMC8404291; doi:10.1186/s13068-021-02023-8)
Supplement: Supplementary file 1 — Additional file 1: Table S1. Primers sequences. Table S2. Identification of metabolites from metabolomics between JW11 strain (A) and JW11 strain cultured with 2% sea salt (B). Table S3. Targeted energy metabolites from LC–MS between JW11 strain (A) and JW11 strain cultured with 2% sea salt (B). Figure S1. Analysis of NADPH level and antioxidative ability of engineered S. elongatus under normal and high salinity stress conditions cultured at days 5, 10 and 15. (A) NADPH level. (B) reactive oxygen species level. (C) Glutathione level. (D) Schematic representation of antioxidative stress system in vivo. Figure S2. Analysis of total fatty acid of engineered S. elongatus under normal and high salinity stress conditions cultured at days 5, 10 and 15. [file 13068_2021_2023_MOESM1_ESM.docx]

**Additional file 1 for**

**Establishment of a resource recycling strategy by optimizing isobutanol production in engineered cyanobacteria** **using high salinity stress**

Xiao-Xi Wu ^a^, Jian-Wei Li ^a^, Su-Fang Xing ^a^, Hui-Ting Chen ^a^, Chao Song ^a^, Shu-Guang Wang ^a^ and Zhen Yan ^a,b,*^

^a^ Shandong Key Laboratory of Water Pollution Control and Resource Reuse, School of Environmental Science and Engineering, Shandong University, Qingdao, Shandong 266237, China

^b^ Suzhou Research Institute, Shandong University, Suzhou, Jiangsu 215123, China

*corresponding author Zhen Yan

E-mail: yanzhen@email.sdu.edu.cn

**Table S1. Primers sequences**

| **Name** | **Sequence (5’-3’)** |
| --- | --- |
| LJW105 | ACAGACCATGGAATTCATGTATACAGTAGGAGATTACCTATTAG |
| LJW106 | CTTTCTCCTTCTAGATTATGATTTATTTTGTTCAGCAAATAGT |
| LJW107 | TCTAGAAGGAGAAAGGTCACATGAACAACTTTAATCTGCACACCCCA |
| LJW108 | CGACTCTAGAGGATCCGCGAAAAGGCCTTTAGCGGGCGGCTTCGTA |
| LJW109 | TTTTTTTGGGCTAGCTTGACAAAAGCAACAAAAGAACAAAAA |
| LJW110 | TGATTCCTCGTCGACCTAGAGAGCTTTCGTTTTCATGAG |
| LJW111 | GTCGACGAGGAATCACCATGGCTAACT |
| LJW112 | ATATCTCCTTCCGGATTAACCCGCAACAGCAATACGT |
| LJW113 | TCCGGAAGGAGATATACCATGCCTAAGTACCGTTC |
| LJW114 | AGCTATGACCATGATTTAACCCCCCAGTTTCGAT |

**Table S2. Identification of metabolites from metabolomics between JW11 strain (A) and JW11 strain cultured with 2% sea salt (B)**

| **adducts** | **Description** | **mzmed** | **rtmed** | **Ratio（B/A）** | **Pvalue（B/A）** | **VIP** |
| --- | --- | --- | --- | --- | --- | --- |
| (M-H)- | gamma-Glutamyl-L-methionine | 277.09 | 526.43 | 0.00 | 0.03 | 1.16 |
| (M-H)- | Sulfaphenazole | 313.08 | 537.48 | 0.01 | 0.04 | 1.12 |
| (M-H)- | gamma-L-Glutamyl-L-phenylalanine | 293.11 | 493.16 | 0.03 | 0.04 | 1.15 |
| (M-H)- | Adenosine monophosphate (AMP) | 346.05 | 626.34 | 0.06 | 0.02 | 1.20 |
| (M-H)- | 1-Palmitoyl-2-oleoyl-phosphatidylglycerol | 747.51 | 61.45 | 0.06 | 0.03 | 1.17 |
| (M-H)- | D-Mannose 1-phosphate | 259.02 | 690.05 | 0.07 | 0.02 | 1.19 |
| (M+CH3COO)- | D-Ribose 5-phosphate | 289.03 | 673.25 | 0.08 | 0.05 | 1.12 |
| (M-H)- | Uridine | 243.06 | 226.51 | 0.15 | 0.00 | 1.30 |
| (M-H)- | Guanosine | 282.08 | 367.34 | 0.33 | 0.03 | 1.18 |
| (2M-H)- | Hydroxyproline | 261.11 | 617.78 | 0.33 | 0.00 | 1.31 |
| (M-H)- | GDP-L-Fucose | 588.07 | 665.71 | 0.36 | 0.00 | 1.35 |
| (M+CH3COO)- | N-Acetylmannosamine | 280.10 | 660.21 | 0.38 | 0.00 | 1.30 |
| (M-H)- | Xanthopterin | 178.04 | 425.41 | 0.39 | 0.03 | 1.17 |
| (M-H)- | Uridine 5'-monophosphate (UMP) | 323.03 | 634.45 | 0.40 | 0.03 | 1.18 |
| (M-H)- | Adenine | 134.05 | 219.91 | 0.42 | 0.00 | 1.30 |
| (M-H)- | 7,8-Dihydrofolate/Succinate | 442.15 | 722.37 | 0.64 | 0.04 | 1.13 |
| (M-H)- | Citrate | 191.02 | 727.18 | 0.73 | 0.01 | 1.26 |
| (M-H2O-H)- | 2-Oxoadipic acid | 141.02 | 574.49 | 2.59 | 0.05 | 1.11 |
| (M-H)- | Glutathione disulfide | 611.15 | 722.50 | 3.49 | 0.02 | 1.21 |
| (M+CH3COO)- | Isobutyrylglycine | 204.09 | 344.67 | 3.65 | 0.00 | 1.33 |
| (M-H)- | Stachyose | 665.21 | 729.02 | 3.82 | 0.00 | 1.33 |
| (M-H)- | Raffinose | 503.16 | 653.73 | 18.72 | 0.00 | 1.30 |
| (M-H)- | Sucrose | 341.11 | 545.89 | 80.02 | 0.00 | 1.34 |
| (M+CH3COO)- | Galactinol | 401.13 | 545.62 | 118.18 | 0.00 | 1.35 |
| (M-H)- | D-Fructose-6-phosphate | 179.05 | 451.51 | 0.42 | 0.04 | 1.08 |
| (M-H2O-H)- | D-Ribulose 5-phosphate | 211.00 | 468.69 | 0.45 | 0.04 | 1.09 |
| (M-H)- | 2,3-Dihydroxy-3-methylbutyric acid | 133.05 | 139.83 | 0.69 | 0.22 | 0.80 |
| (M-H)- | 4-Hydroxybenzoate | 137.02 | 193.51 | 0.40 | 0.13 | 0.94 |
| (M-H)- | 5-L-Glutamyl-L-alanine | 217.08 | 605.50 | 2.05 | 0.06 | 1.08 |
| (M-H2O-H)- | alpha-D-Galactose 1-phosphate | 241.01 | 465.79 | 1.10 | 0.52 | 0.45 |
| (M-H)- | Azelaic acid | 187.10 | 359.78 | 1.16 | 0.47 | 0.49 |
| (M-H)- | BHT | 219.17 | 317.22 | 0.91 | 0.44 | 0.53 |
| (M-H)- | Cytidine | 242.08 | 341.40 | 0.77 | 0.18 | 0.86 |
| (M-H)- | gamma-L-Glutamyl-L-glutamic acid | 275.09 | 679.70 | 0.79 | 0.28 | 0.71 |
| (M-H)- | gamma-L-Glutamyl-L-valine | 245.11 | 543.88 | 0.01 | 0.06 | 1.08 |
| (M-H)- | L-Glutamate | 146.04 | 593.32 | 1.35 | 0.66 | 0.32 |
| (M-H)- | L-Isoleucine | 130.09 | 381.04 | 1.52 | 0.64 | 0.32 |
| (M-H)- | L-Phenylalanine | 164.07 | 365.22 | 0.57 | 0.43 | 0.54 |
| (M-H)- | L-Pyroglutamic acid | 128.03 | 420.83 | 1.51 | 0.23 | 0.77 |
| (M-H)- | L-Tryptophan | 203.08 | 364.90 | 0.50 | 0.25 | 0.76 |
| (M-H)- | Maltotriose | 503.16 | 691.68 | 2.57 | 0.18 | 0.85 |
| (M-H)- | Mupirocin | 499.30 | 33.36 | 1.37 | 0.46 | 0.53 |
| (M-H)- | Norethindrone Acetate | 339.20 | 34.66 | 1.75 | 0.52 | 0.45 |
| (M-H)- | Quinate | 191.05 | 477.94 | 15.96 | 0.15 | 0.91 |
| (M-H)- | Thymidine | 241.08 | 72.87 | 0.52 | 0.06 | 1.08 |
| (M-H)- | UDP-D-Galactose | 565.04 | 650.39 | 0.67 | 0.32 | 0.66 |
| (M-H)- | UDP-N-acetylglucosamine | 606.06 | 631.44 | 1.52 | 0.18 | 0.86 |
| (M-H)- | UDP-N-acetylmuraminate | 678.09 | 650.92 | 0.15 | 0.05 | 1.10 |
| (2M+H) + | (R)-3-Hydroxybutyric acid | 209.10 | 367.83 | 15.46 | 0.01 | 1.20 |
| (M+H-H2O) + | 1,2-Benzenedicarboxylic acid | 149.02 | 46.71 | 1.14 | 0.57 | 0.38 |
| (M+H) + | 1-Oleoyl-sn-glycero-3-phosphocholine | 522.35 | 274.66 | 1.94 | 0.33 | 0.62 |
| (M+H-H2O) + | 1-Palmitoylglycerol | 313.27 | 163.43 | 0.04 | 0.00 | 1.26 |
| (M+H) + | 1-Palmitoyl-sn-glycero-3-phosphocholine | 496.34 | 65.13 | 3.19 | 0.12 | 0.89 |
| (M+H-H2O) + | 1-Stearoyl-sn-glycerol | 341.30 | 61.84 | 0.19 | 0.01 | 1.20 |
| (2M+Na) + | 2-Amino-1-phenylethanol | 297.15 | 341.16 | 2.12 | 0.24 | 0.73 |
| (M+CH3COO+2H) + | 2-Ethoxyethanol | 151.10 | 71.17 | 3.45 | 0.06 | 1.03 |
| (M+H) + | 2-Hydroxyadenine | 152.06 | 323.15 | 2.62 | 0.01 | 1.20 |
| (2M+NH4) + | 3,4-Dimethoxycinnamic acid | 434.19 | 656.89 | 0.27 | 0.05 | 1.05 |
| (M+H-H2O) + | 6-Aminocaproic acid | 114.09 | 64.52 | 3.08 | 0.17 | 0.82 |
| (M+H-H2O) + | 7,8-Dihydrobiopterin | 222.10 | 147.23 | 0.02 | 0.09 | 0.96 |
| (M+H) + | Acetylcarnitine | 204.12 | 457.65 | 908.18 | 0.36 | 0.58 |
| (M+H) + | Adenosine | 268.10 | 242.71 | 1.48 | 0.58 | 0.37 |
| M+ | Bata-Carotene | 536.44 | 45.99 | 0.06 | 0.00 | 1.26 |
| (M+H) + | Betaine | 118.09 | 825.67 | 1.34 | 0.45 | 0.49 |
| (M+H-H2O) + | Biopterin | 220.08 | 197.30 | 0.04 | 0.01 | 1.19 |
| M+ | Choline | 104.11 | 422.55 | 36.89 | 0.00 | 1.22 |
| (M+H-H2O) + | cis-9-Palmitoleic acid | 237.22 | 212.90 | 0.03 | 0.11 | 0.92 |
| (M+CH3COO+2H) + | Cyclohexylamine | 160.13 | 590.11 | 0.05 | 0.00 | 1.23 |
| (M+H) + | Cytidine 5'-monophosphate (CMP) | 324.06 | 657.48 | 0.04 | 0.00 | 1.22 |
| (M+H) + | Deoxyadenosine | 252.11 | 182.66 | 0.78 | 0.60 | 0.35 |
| (M+H) + | Deoxycytidine | 228.10 | 294.15 | 0.09 | 0.03 | 1.08 |
| (M+H) + | Dioctyl phthalate | 391.28 | 46.53 | 0.36 | 0.02 | 1.14 |
| (M+H-H2O) + | DL-Indole-3-lactic acid | 188.07 | 363.99 | 0.62 | 0.44 | 0.50 |
| (M+H) + | D-Mannose-6-phosphate | 261.04 | 690.29 | 0.08 | 0.01 | 1.19 |
| (M+H) + | D-Proline | 116.07 | 451.86 | 8.98 | 0.06 | 1.01 |
| (M+H) + | EDTA | 293.10 | 631.70 | 20.49 | 0.23 | 0.73 |
| (M+H) + | Erucamide | 338.34 | 49.89 | 0.08 | 0.02 | 1.13 |
| (M+H) + | Glu-Ser | 235.09 | 635.01 | 0.26 | 0.02 | 1.15 |
| (M+H) + | Glycerol 1-myristate | 303.25 | 166.34 | 0.13 | 0.00 | 1.27 |
| (M+H) + | Ile-Glu | 261.14 | 526.40 | 0.00 | 0.00 | 1.27 |
| (M+H) + | L-Norleucine | 132.10 | 376.23 | 4.03 | 0.27 | 0.69 |
| (M+H) + | L-Tyrosine | 182.08 | 439.77 | 1.07 | 0.85 | 0.13 |
| (M+NH4) + | Maltopentaose | 846.31 | 733.52 | 7.68 | 0.00 | 1.27 |
| (M+CH3CN+H) + | Met-Gln | 319.15 | 523.92 | 0.03 | 0.00 | 1.23 |
| (M+H) + | Methoprene (S) | 311.26 | 60.53 | 0.17 | 0.00 | 1.26 |
| (M+H) + | N6,N6,N6-Trimethyl-L-lysine | 189.16 | 788.50 | 2.69 | 0.04 | 1.08 |
| (M+CH3COO+2H) + | N-Acetylglutamine | 249.11 | 614.47 | 0.36 | 0.06 | 1.01 |
| (M+H) + | NG,NG-dimethyl-L-arginine(ADMA) | 203.15 | 733.15 | 8.29 | 0.00 | 1.24 |
| (M+H) + | Nicotinamide | 123.05 | 104.70 | 0.47 | 0.04 | 1.06 |
| (M+H) + | Nicotinamide adenine dinucleotide (NAD) | 664.11 | 645.90 | 5.12 | 0.01 | 1.17 |
| (M+H) + | Nicotine | 163.12 | 141.39 | 23.55 | 0.39 | 0.56 |
| (M+CH3CN+H) + | Pelletierine | 183.15 | 471.08 | 2.81 | 0.27 | 0.69 |
| (M+H) + | Phosphorylcholine | 184.07 | 728.82 | 4.45 | 0.22 | 0.75 |
| (M+H) + | Phthalic acid Mono-2-ethylhexyl Ester | 279.16 | 46.70 | 1.70 | 0.02 | 1.14 |
| (M+NH4) + | Phytanic acid | 330.34 | 64.72 | 0.14 | 0.11 | 0.91 |
| (M+H) + | Pro-Gly | 173.09 | 173.82 | 25.76 | 0.19 | 0.80 |
| (M+H) + | S-Methyl-5'-thioadenosine | 298.10 | 156.62 | 2.62 | 0.01 | 1.16 |
| (M+H-2H2O) + | Tauroursodeoxycholic acid | 464.28 | 65.44 | 8.11 | 0.19 | 0.80 |
| (M+Na) + | Thioetheramide-PC | 758.57 | 206.69 | 0.12 | 0.40 | 0.54 |
| (M+H) + | Thymine | 127.05 | 73.29 | 2.62 | 0.00 | 1.26 |
| (M+CH3COO+2H) + | trans,trans-Farnesol | 283.23 | 60.94 | 0.12 | 0.00 | 1.23 |
| (M+H) + | Triethanolamine | 150.11 | 248.70 | 1.91 | 0.19 | 0.79 |
| (M+H-H2O) + | Tyramine | 120.08 | 366.80 | 1.23 | 0.69 | 0.27 |
| (M+H) + | Tyr-Glu | 311.12 | 559.59 | 0.00 | 0.02 | 1.15 |
| (M+CH3COO+2H) + | Tyr-Gly | 299.13 | 135.03 | 2.58 | 0.08 | 0.98 |

**Table S3. Targeted energy metabolites from LC-MS between JW11 strain (A) and JW11 strain cultured with 2% sea salt (B)**

| **Detection object** | **Precursor Mz** | **Product Mz** | **Retention Time** | **Area** |
| --- | --- | --- | --- | --- |
| 3-phosphoglycerate | 185 | 97 | A1：11.58 | 1141457 |
|  |  |  | A2：11.16 | 607734 |
|  |  |  | A3：11.35 | 668774 |
|  |  |  | B1：11.17 | 699989 |
|  |  |  | B2：11.21 | 349417 |
|  |  |  | B3：11.45 | 365219 |
| Aconitate | 173 | 129.01 | A1：8.44 | 69080 |
|  |  |  | A2：8.27 | 145961 |
|  |  |  | A3：8.06 | 2175152 |
|  |  |  | B1：7.42 | 244256 |
|  |  |  | B2：7.03 | 41085 |
|  |  |  | B3：8.06 | 60772 |
| ADP | 426 | 134 | A1：6.65 | 3630 |
|  |  |  | A2：6.81 | 5290 |
|  |  |  | A3：6.73 | 3857 |
|  |  |  | B1：6.76 | 2975 |
|  |  |  | B2：6.69 | 4015 |
|  |  |  | B3：6.73 | 3196 |
| ADPglucose | 588 | 346 | A1：10.81 | 22713 |
|  |  |  | A2：10.80 | 45650 |
|  |  |  | A3：10.77 | 23430 |
|  |  |  | B1：10.49 | 1984 |
|  |  |  | B2：10.41 | 1708 |
|  |  |  | B3：10.51 | 1430 |
| a-Ketoglutarate | 145 | 101 | A1：8.99 | 158267 |
|  |  |  | A2：8.99 | 201686 |
|  |  |  | A3：8.76 | 436714 |
|  |  |  | B1：8.71 | 113560 |
|  |  |  | B2：8.71 | 53020 |
|  |  |  | B3：8.68 | 51645 |
| alpha-D-Ribose 5-phosphate | 229 | 79 | A1：11.67 | 240736 |
|  |  |  | A2：11.48 | 665119 |
|  |  |  | A3：11.54 | 1085149 |
|  |  |  | B1：12.15 | 75043 |
|  |  |  | B2：12.44 | 28765 |
|  |  |  | B3：12.09 | 25135 |
| AMP | 346 | 79 | A1：11.36 | 3964147 |
|  |  |  | A2：11.30 | 6274105 |
|  |  |  | A3：11.31 | 6532551 |
|  |  |  | B1：11.49 | 549068 |
|  |  |  | B2：11.81 | 462223 |
|  |  |  | B3：11.48 | 216809 |
| cAMP | 328 | 134 | A1：6.49 | 50469 |
|  |  |  | A2：6.49 | 10689 |
|  |  |  | A3：6.51 | 121874 |
|  |  |  | B1：6.84 | 37962 |
|  |  |  | B2：6.85 | 51040 |
|  |  |  | B3：6.93 | 27170 |
| D-Fructose 1,6-bisphosphate | 339 | 97 | A1：15.29 | 199871 |
|  |  |  | A2：15.47 | 186836 |
|  |  |  | A3：15.39 | 138805 |
|  |  |  | B1：15.34 | 110321 |
|  |  |  | B2：15.81 | 115432 |
|  |  |  | B3：15.23 | 143256 |
| D-Glucose 1-phosphate | 259 | 241 | A1：12.89 | 936270 |
|  |  |  | A2：12.22 | 342762 |
|  |  |  | A3：12.69 | 836371 |
|  |  |  | B1：12.27 | 1890878 |
|  |  |  | B2：12.79 | 2885426 |
|  |  |  | B3：12.81 | 6356939 |
| Dihydroxyacetone phosphate | 169 | 97.01 | A1：2.17 | 360628 |
|  |  |  | A2：2.01 | 160837 |
|  |  |  | A3：2.49 | 592954 |
|  |  |  | B1：1.86 | 343747 |
|  |  |  | B2：1.92 | 1062653 |
|  |  |  | B3：2.07 | 229712 |
| FMN | 455 | 213 | A1：10.86 | 28100 |
|  |  |  | A2：10.83 | 38555 |
|  |  |  | A3：10.87 | 51700 |
|  |  |  | B1：10.81 | 15290 |
|  |  |  | B2：10.81 | 20955 |
|  |  |  | B3：10.52 | 19745 |
| Fumarate | 115 | 71 | A1：9.02 | 230308 |
|  |  |  | A2：9.45 | 215271 |
|  |  |  | A3：9.47 | 344036 |
|  |  |  | B1：9.03 | 25740 |
|  |  |  | B2：9.01 | 5720 |
|  |  |  | B3：9.00 | 2915 |
| Glyceraldehyde 3-phosphate | 169 | 97.02 | A1：2.17 | 381095 |
|  |  |  | A2：2.01 | 260055 |
|  |  |  | A3：2.49 | 618195 |
|  |  |  | B1：1.86 | 254561 |
|  |  |  | B2：1.92 | 325412 |
|  |  |  | B3：2.07 | 248007 |
| Isocitrate | 191 | 73 | A1：10.73 | 27260 |
|  |  |  | A2：10.45 | 27830 |
|  |  |  | A3：10.75 | 30085 |
|  |  |  | B1：10.97 | 19580 |
|  |  |  | B2：10.48 | 21615 |
|  |  |  | B3：10.58 | 24970 |
| L-Lactate | 89 | 45 | A1：5.12 | 409257 |
|  |  |  | A2：4.97 | 615747 |
|  |  |  | A3：5.15 | 678617 |
|  |  |  | B1：5.33 | 36960 |
|  |  |  | B2：4.66 | 7163 |
|  |  |  | B3：5.47 | 11461 |
| L-Malic acid | 133 | 115 | A1：9.34 | 131341 |
|  |  |  | A2：9.91 | 311003 |
|  |  |  | A3：9.95 | 2014551 |
|  |  |  | B1：8.77 | 76945 |
|  |  |  | B2：8.55 | 12210 |
|  |  |  | B3：8.93 | 42460 |
| NAD | 662 | 540 | A1：10.77 | 615013 |
|  |  |  | A2：10.70 | 385607 |
|  |  |  | A3：10.76 | 649114 |
|  |  |  | B1：10.84 | 16805 |
|  |  |  | B2：10.93 | 15980 |
|  |  |  | B3：10.95 | 1870 |
| NADH | 664 | 408 | A1：9.82 | 892 |
|  |  |  | A2：10.18 | 465 |
|  |  |  | A3：9.59 | 715 |
|  |  |  | B1：9.60 | 1595 |
|  |  |  | B2：9.68 | 1440 |
|  |  |  | B3：9.71 | 2070 |
| Phosphoenolpyruvate | 167 | 79 | A1：11.76 | 200806 |
|  |  |  | A2：11.73 | 204436 |
|  |  |  | A3：11.49 | 317507 |
|  |  |  | B1：12.04 | 71745 |
|  |  |  | B2：12.20 | 101941 |
|  |  |  | B3：12.66 | 107900 |
| Pyruvate | 87 | 43 | A1：2.05 | 428777 |
|  |  |  | A2：1.99 | 453973 |
|  |  |  | A3：2.33 | 357743 |
|  |  |  | B1：2.22 | 684584 |
|  |  |  | B2：2.16 | 741927 |
|  |  |  | B3：2.33 | 799974 |
| Succinate | 117 | 73 | A1：5.78 | 12395541 |
|  |  |  | A2：5.74 | 26468108 |
|  |  |  | A3：5.74 | 47182280 |
|  |  |  | B1：7.69 | 2257708 |
|  |  |  | B2：6.39 | 81659 |
|  |  |  | B3：6.38 | 47882 |
| Thiamine pyrophosphate (TPP) | 423.1 | 302 | A1：14.99 | 58410 |
|  |  |  | A2：15.48 | 52343 |
|  |  |  | A3：14.78 | 48620 |
|  |  |  | B1：15.16 | 65518 |
|  |  |  | B2：15.22 | 28600 |
|  |  |  | B3：15.09 | 27882 |
| UDPglucose | 565 | 323 | A1：10.88 | 3173463 |
|  |  |  | A2：10.78 | 5136480 |
|  |  |  | A3：10.89 | 3005767 |
|  |  |  | B1：10.93 | 34430 |
|  |  |  | B2：10.59 | 15950 |
|  |  |  | B3：10.56 | 23868 |
| Acetyl-CoA | 810.1 | 303.1 | A1：11.12 | 30304 |
|  |  |  | A2：10.68 | 42020 |
|  |  |  | A3：10.62 | 50105 |
|  |  |  | B1：11.58 | 7040 |
|  |  |  | B2：11.90 | 5290 |
|  |  |  | B3：10.12 | 2590 |
| GMP | 364 | 152.1 | A1：12.08 | 464148 |
|  |  |  | A2：11.88 | 801684 |
|  |  |  | A3：11.90 | 848117 |
|  |  |  | B1：12.92 | 132964 |
|  |  |  | B2：13.56 | 125016 |
|  |  |  | B3：13.49 | 46750 |
| L-Glutamate | 148.1 | 84.1 | A1：9.95 | 30221626 |
|  |  |  | A2：9.90 | 37984548 |
|  |  |  | A3：9.96 | 32329846 |
|  |  |  | B1：10.01 | 14505910 |
|  |  |  | B2：10.04 | 10206517 |
|  |  |  | B3：10.05 | 16434327 |
| L-Glutamine | 147 | 84.1 | A1：9.58 | 1119864 |
|  |  |  | A2：9.55 | 1496395 |
|  |  |  | A3：9.59 | 3108653 |
|  |  |  | B1：9.79 | 136477 |
|  |  |  | B2：9.78 | 138750 |
|  |  |  | B3：9.76 | 200916 |
| NADP | 742 | 620 | A1：11.86 | 1141457 |
|  |  |  | A2：11.95 | 607734 |
|  |  |  | A3：11.95 | 668774 |
|  |  |  | B1：11.97 | 699989 |
|  |  |  | B2：11.58 | 349417 |
|  |  |  | B3：11.93 | 365219 |
| NADPH | 744 | 408 | A1：11.29 | 69080 |
|  |  |  | A2：11.15 | 145961 |
|  |  |  | A3：11.19 | 2175152 |
|  |  |  | B1：11.26 | 244256 |
|  |  |  | B2：11.24 | 41085 |
|  |  |  | B3：11.29 | 60772 |


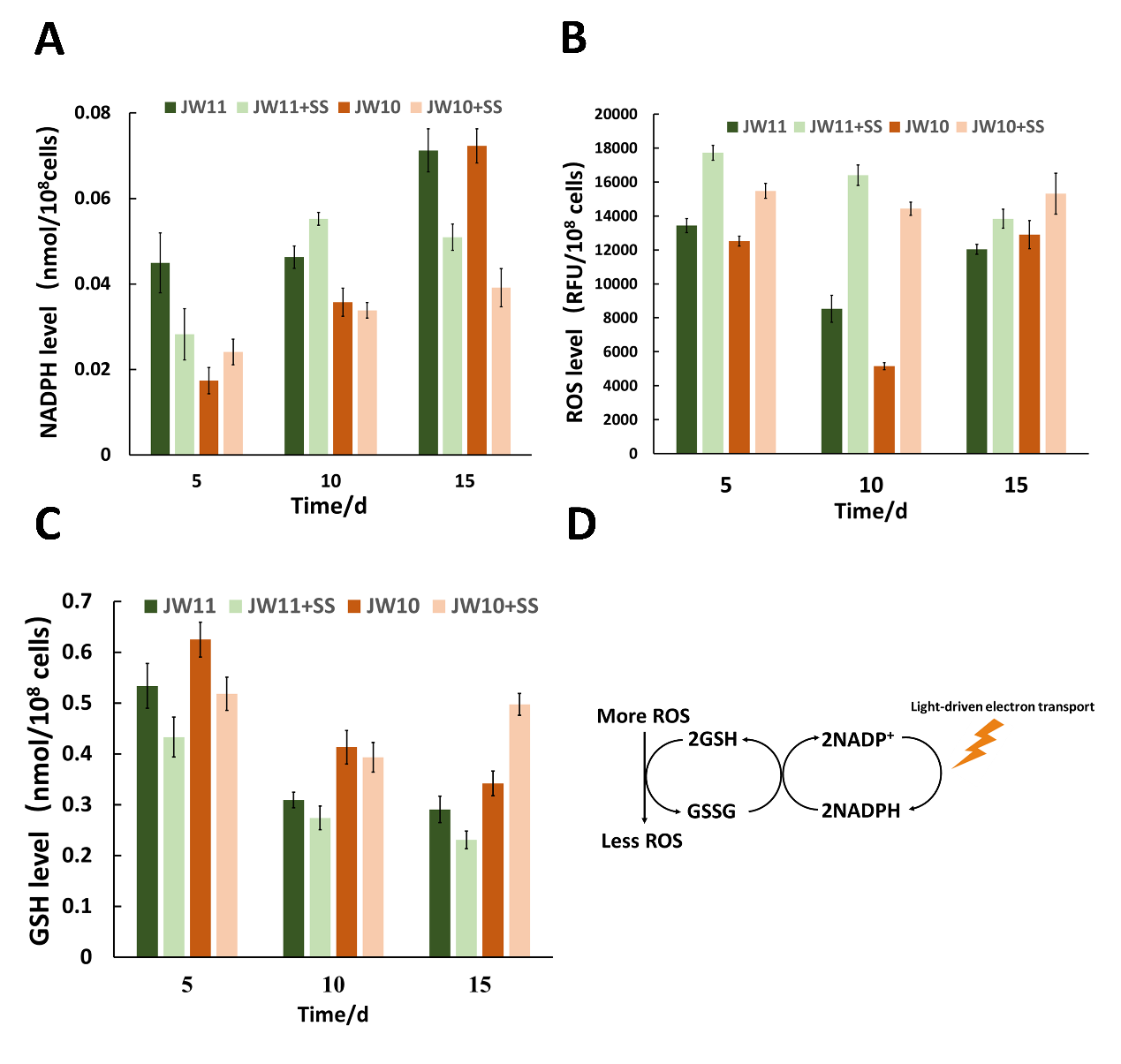


**Fig. S1. Analysis of NADPH level and antioxidative ability of engineered *S. elongatus* under normal and high salinity stress conditions cultured at day 5, 10 and 15.** (A) NADPH level. (B) reactive oxygen species level. (C) Glutathione level. (D) Schematic representation of antioxidative stress system in vivo.

**
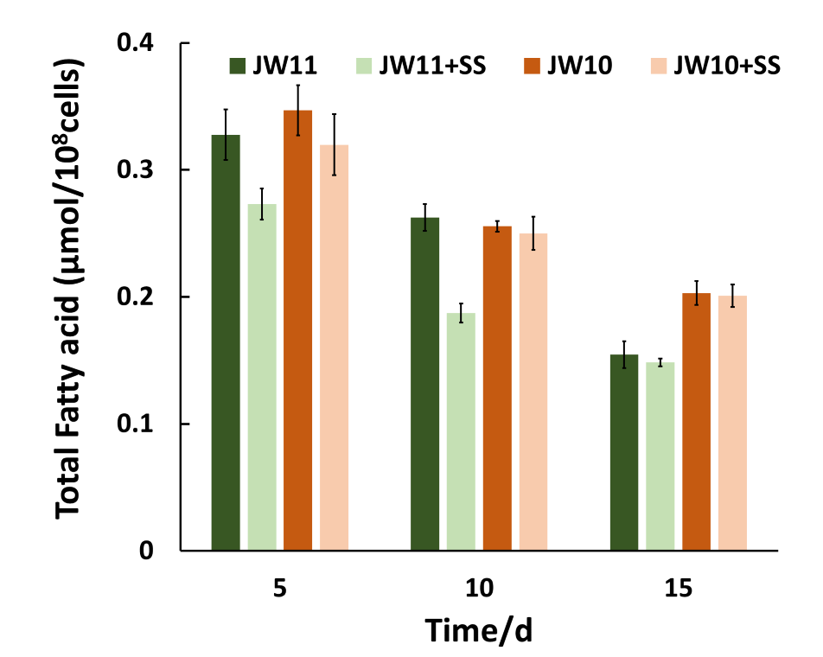
**

**Fig. S2. Analysis of total fatty acid of engineered *S. elongatus* under normal and high salinity stress conditions cultured at day 5, 10 and 15.**
